# Educational inequalities in self-reported health in a general Iranian population

**DOI:** 10.1186/1756-0500-1-50

**Published:** 2008-07-21

**Authors:** Ali Montazeri, Azita Goshtasebi, Mariam Vahdaninia

**Affiliations:** 1 Iranian Institute for Health Sciences Research, Tehran, Iran

## Abstract

**Background:**

The aim of this study was to investigate the relationship between educational level and self-reported health in an Iranian population, in order to provide evidence on social inequalities in health from a country in which such data need to be collected.

**Methods:**

This population-based study was carried out in Tehran, Iran. Individuals aged 15 years and over were interviewed. Self-reported health was measured by asking each individual to respond to the question: "In general how would you describe your health at present?" We used years of formal education as a measure of socioeconomic status and categorized the answers in five levels. Logistic regression analysis was used to estimate odds ratios and 95% confidence intervals indicating the contribution of educational level to self-reported health, adjusting for age, gender, marital status, and chronic diseases.

**Results:**

In all, 4163 individuals were interviewed. The mean age of the respondents was 35.1 years (SD = 16.0); 52% were female; the mean duration of formal education was 10.0 years (SD = 4.5); and 31% rated their health 'less than good'. Overall, women rated their health more poorly than men (P < 0.0001), and the findings showed that those with higher education rated their health significantly better than those with lower educational levels after adjusting for the age, gender, marital status and chronic diseases. The odds ratio for having 'less than good' self-rated health in those at the lowest educational level compared with those at the highest was 2.65 (95% CI = 1.88–3.73).

**Conclusion:**

The findings indicated an inverse relationship between educational level and self-rated health, and that age, gender, and chronic conditions had independent effects on self-reported health status. The findings of this first study from Iran suggest that health inequalities in developing countries such as Iran need to be addressed and policies for tackling the problem should be considered. In this respect, less well-educated people and women should be seen as the first target populations. It seems that although expanding the educational system might help the state to provide people with more educational options, it is also necessary to ensure that equal opportunities and access to quality education are provided for those from lower socioeconomic backgrounds; otherwise the current situation might cost the government more in the long term because of poor health among disadvantaged groups.

## Background

In recent years, compelling evidence has been obtained for an inverse relationship between health and socioeconomic status over time and in different countries [1,2]. This association has been found for all indicators of socioeconomic level whether they are based on occupation, education or income [3,4]. Studies have shown that socioeconomic levels have both direct and indirect effects on health [5]. However, the magnitude of health disparities across socioeconomic levels varies within and between countries [6]. It has been suggested that reducing health inequalities in disadvantaged groups may offer great potential for improving the health status of the population as a whole [7]. Thus, the World Health Organization now considers the reduction of health inequalities to be one of the top priorities [8].

The aim of this study was to describe self-reported health by educational level in an Iranian population. It is believed that self-rated health is a valuable measure in health-related inequality research because it is based on individuals' own assessments of the trajectories of their social and familial histories and on how they perceive their health status, and it reflects the availability of resources and environmental factor that may ultimately affect health [3,9,10]. We believe the same argument may apply to the general public in Iran, so the findings from this study might be a good starting point for future research on the topic here and in other developing countries in Asia. In addition, Iran has a complex educational system and educational attainment could reflect individuals' socioeconomic positions. Therefore our hypothesis was that educational achievements contribute to self-rated health. To our knowledge, this is the first paper from Iran that reports on the topic. It may therefore add to the existing evidence on international variations in socioeconomic inequalities in self-reported health. It may also facilitate the reduction of health inequalities in the population by raising awareness among research communities and providing evidence for policymakers, affecting national level policies, though at present there are no such policies in force in Iran. In Europe there are national level policies to promote health equity within and between the countries [11]. It has been suggested that information and knowledge-sharing has a key role in linking evidence about the social and environmental causes of health inequalities to local actions and challenges [12].

## Methods

### Design

This study was based on information taken from a cross-sectional population-based survey on quality of life carried out in Tehran, Iran. To select a representative sample of the general population aged 15 years and over a stratified multi-stage area sampling was applied. Every household within 22 different districts in Tehran had the same probability of being sampled. For the first stage, units (blocks) were randomly selected after stratifying by district and size of residence. Then the homes to be sampled within each block were selected by random routes. Finally, the last-stage sampling units (the individuals) were selected randomly from all persons living in the same home.

### Self-reported health

Self-reported health was measured by asking each individual to respond to the question: "In general how would you describe your health at present?" There were five response categories; 'excellent', 'very good', 'good', 'fair', and 'poor'. For analysis we combined the categories 'excellent', 'very good' and 'good' to yield a measure of self-reported health of 'good or better than good' and the categories 'fair' and 'poor' to yield a measure of 'less than good'. There is evidence that using a general question on self-reported health measured on an ordinal scale is a valid instrument for determining individuals' perceptions in studies of health inequalities [12].

### Education

We used years of formal education as a measure of socioeconomic status. It is argued that educational level is a variable that can be applied to the entire population [13]. It has been shown that stratification by education is probably the best measure of socioeconomic status when results from different populations are compared [4,14]. In addition, since income information in Iran is not reliable and people usually have more than one job at the same time, we did not collect or use data on income or occupation as a measure of socioeconomic position. However, education was categorized into five levels: no education, first level (1 to 5 years), second level (6–9 years), third level (10–12 years) and fourth level (more than 12 years).

In principle, everybody in Iran should have free access to education even at higher levels. However, since Iran has a young population (50% under 30 years old) and the state could not respond to the potential need, methods of payments for education currently vary: completely free of charge, partially paid and completely paid. All these are governed by the state and overall a unique formal system is followed: primary education (1–5 years), high school (6–9 and 10–12 years), and college (13–14 years)/or university (13–16 years and more). The success rate for entering higher education is usually high for those who can afford paid education (either partially or completely paid).

### Chronic diseases

Data on chronic diseases were collected by asking each respondent to indicate whether he or she suffered from any medically diagnosed chronic condition including cardiovascular diseases, diabetes, musculoskeletal disorders, cancers, neurological or psychological diseases and chronic respiratory diseases.

### Statistical analysis

The potential variation in self-reported health by educational level was estimated by odds ratios, using logistic regression and adjusting for age, gender, and chronic conditions. We also entered martial status into the logistic regression model since there were significant differences between men and women with regard to marital status. Self-reported excellent/very good/good health (i.e. good or better than good) versus fair/poor health (i.e. less than good) was the comparison [3].

### Ethics

The study received ethical approval from the Iranian Institute for Health Sciences Research and all participants gave oral consent.

## Results

In all, 4804 individuals were approached and 4163 (87%) agreed to be interviewed. Of those who did not participate in the study, 230 were female and the remaining 411 were male. The main reason for non-participation was that after two approaches most of these individuals were not available in their homes. Only a few refused to take part in the study because of dislike (n = 64). The characteristics of the study sample are shown in Table 1. The mean age of the respondents was 35.1 (SD = 16.0) years. Fifty-two percent were female, mostly married (58%), and the mean duration of formal education for the whole study sample was 10.0 years (SD = 4.5). Sixty-nine percent of the respondents rated their health 'good or better than good' (excellent, very good, or good), while 31% rated their health 'less than good' (fair or poor). Seven percent of the respondents (n = 296) indicated that they suffered from a medically diagnosed chronic condition.

**Table 1:** The characteristics of the study sample

	**All (n = 4163)**	**Male (n = 1997)**	**Female (n = 2166)**	**P**
	**No. (%)**	**No. (%)%**	**No. (%)**	

**Age group**				< 0.0001
15–24	1420 (34)	681 (34)	739 (34)	
25–44	1614 (39)	721 (36)	893 (41)	
45–64	882 (21)	446 (22)	436 (20)	
≥ 65	247 (6)	149 (8)	98 (5)	
Mean (SD)	35.1 (16.0)	36.1 (16.9)	34.1 (15.1)	< 0.0001
**Marital status**				< 0.0001
Single	1601 (38)	827 (47)	774 (36)	
Married	2406 (58)	1149 (52)	1257 (58)	
Widowed/divorced	156 (4)	21 (1)	135 (6)	
**Educational level (years)**				< 0.0001
No education	280 (7)	100 (5)	180 (8)	
First level (1–5)	475 (11)	211 (11)	264 (12)	
Second level (6–9)	901 (22)	460 (23)	441 (21)	
Third level (10–12)	1695 (41)	783 (39)	912 (42)	
Fourth level (>12)	812 (19)	443 (22)	369 (17)	
Mean (SD)	10.0 (4.5)	10.4 (4.3)	9.6 (4.5)	< 0.0001
**Self-reported health**				< 0.0001
Excellent/very good	1450 (35)	803 (40)	647 (30)	
Good	1405 (34)	655 (33)	750 (34)	
Fair/poor	1308 (31)	539 (27)	769 (36)	
**Chronic diseases**				0.004
Yes	296 (7)	117 (6)	179 (8)	
No	3867 (93)	1880 (94)	1987 (92)	
**Diseases **(n = 296)				0.01
Hypertension	31 (11)	7 (6)	24 (13)	
Other cardiovascular diseases	78 (26)	43 (37)	35 (20)	
Diabetes	22 (7)	8 (7)	14 (8)	
Musculoskeletal disorders	95 (32)	34 (29)	61 (34)	
Cancer	5 (2)	0 (0)	5 (3)	
Neurological and psycho-logical diseases	45 (15)	19 (16)	26 (15)	
Chronic respiratory diseases	20 (7)	6 (5)	14 (8)	

As indicated in Table 1, women rated their health more poorly than men (χ^2 ^= 56.9, df = 2, P < 0.0001), were younger (mean age 36.1 vs. 34.2 years, P < 0.0001) and were less educated than men (χ^2 ^= 38.9, df = 4, P < 0.0001). There were also significant differences between men and women in having chronic conditions (χ^2 ^= 16.4, df = 6, P = 0.01).

The most important findings of the study are shown in Table 2. There was a significant association between educational level and self-reported health: those with higher education rated their health significantly better than those with lower educational levels. The odds ratio for having 'less than good' self-reported health for those with the lowest educational level compared with those with the highest was 2.65 (95% CI = 1.88–3.73).

**Table 2:** The odds ratio for 'less than good' self-rated health obtained from logistic regression analysis on all the study sample, on men and on women

	**OR**	**95% CI**	**P**
**All (n = 4163)**			
*Age*	1.05	1.04–1.06	< 0.0001
*Gender*			
Male	1.0 (ref.)		
Female	1.65	1.41–1.93	< 0.0001
*Marital status*			
Single	1.0 (ref.)		
Married	1.57	1.25–1.96	< 0.0001
Widowed/divorced	2.33	1.45–3.73	< 0.0001
*Chronic diseases*			
No	1.0 (ref.)		
Yes	2.72	2.03–3.65	< 0.0001
*Educational level (years)*			
Fourth level (> 12)	1.0 (ref.)		
Third level (10–12)	1.56	1.25–1.94	< 0.0001
Second level (6–9)	1.85	1.45–2.35	< 0.0001
First level (1–5)	1.86	1.42–2.43	< 0.0001
No education	2.65	1.88–3.73	< 0.0001
**Male (n = 1997)**			
*Age*	1.05	1.04–1.06	< 0.0001
*Marital status*			
Single	1.0 (ref.)		
Married	1.27	0.90–1.79	0.17
Widowed/divorced	8.28	2.52–27.2	0.001
*Chronic diseases*			
No	1.0 (ref.)		
Yes	2.44	1.58–3.78	< 0.0001
*Educational level (years)*			
Fourth level (> 12)	1.0 (ref.)		
Third level (10–12)	1.26	0.92–1.72	0.14
Second level (6–9)	1.43	1.02–1.98	0.03
First level (1–5)	1.62	1.10–2.39	0.01
No education	3.07	1.80–5.24	< 0.0001
**Female (n = 2166)**			
*Age*	1.05	1.04–1.06	< 0.0001
*Marital status*			
Single	1.0 (ref.)		
Married	1.72	1.28–2.32	< 0.0001
Widowed/divorced	1.91	1.09–3.35	0.02
*Chronic diseases*			
No	1.0 (ref.)		
Yes	2.99	2.00–4.48	< 0.0001
*Educational level (years)*			
Fourth level (> 12)	1.0 (ref.)		
Third level (10–12)	1.91	1.38–2.63	< 0.0001
Second level (6–9)	2.35	1.65–3.34	< 0.0001
First level (1–5)	2.13	1.44–3.14	< 0.0001
No education	2.60	1.63–4.12	< 0.0001

Separate analysis of the data for males and females showed different pictures for the variables studied. Poorer self-reported health in men was not significantly associated with marriage or third level education (10–12 years); for women poorer self-reported health was strongly associated with all levels of education and marital status. However, the contribution of education to 'less than good' self-rated health was simultaneously more gradual and steeper in males than in females (Table 2).

## Discussion

This was a population-based study of the relationship between educational level and self-reported health in Tehran, Iran. There was a distinct pattern of self-reported health among those with different educational levels, showing a dose-response relationship between education and the risk of 'less than good' self-rated health status. However, one might argue that a sample from the urban capital (Tehran) is not necessarily representative of the entire country. In general this is true, but since Tehran has became a multicultural metropolitan area with a mixture of different socioeconomic and ethnic backgrounds, a sample from the general population in Tehran could at least be regarded as representative of the urban population of Iran [15]. It has been suggested that studies of self-reported health including quality of life assessments provide information on the health of a population that are usually invisible in traditional analyses of population health [16]. Responses to questions of this type (self-reported health or self-reported morbidity) may vary according to mode of administration, but studies have shown that such responses are nevertheless valid [17].

The findings from the present study indicate that in general people with a higher educational level rated their health status more highly than people with a lower educational level. There are several explanations for education-related health inequalities within and between countries. The most straightforward is that the effect of education varies from one place to another for unknown reasons, which would make it difficult to account for differences in the magnitude of the association between education and various health indicators in different countries [18]. In addition, international studies have shown that educational health inequalities vary in magnitude between countries [19]. A recent publication from the Eurothine Project [20], which compares inequalities in health in more than 20 European countries, suggests that although income and education are 'upstream' determinants of health inequalities, this is the feature of European welfare regimes that could provide evidence for the magnitudes of educational health inequalities between countries. They found that South European welfare regimes had the largest health inequalities while countries with Bismarckian welfare regimes tended to have the smallest. Although the other welfare regimes ranked relatively close to each other, the Scandinavian regimes were placed less favorably than the Anglo-Saxon and East European [21].

Another possible explanation is that in societies where everyone has the same access to education, educational level is not a good indicator of socioeconomic position. Thus, even if there is an inverse relationship between education and self-reported health, this does not demonstrate inequality in health in such societies [4]. On the other hand, it can be argued educational attainment could reflect dissimilarities among individuals in terms of work conditions, economic status, lifestyle and the use of health care services, so education has a significant impact on the observed inequalities in health among different socioeconomic subgroups of populations [22]. Others have suggested that educational inequalities in health might be attributable to the fact that education reflects the different life course accumulations of material and psychosocial hazards to which people have been exposed [23]. Since not everyone has the same access to education in Iran, we suspect that the latter explanation applies to the variation in self-reported health by educational level. Interestingly, a study using data from the 2003 US Current Population Health Survey indicated that people in the very high income bracket tend to report slightly worse health, which may be explained by their lower education [24].

We found that women rated their health more poorly than men. This was definitely not due to age since women were younger than men on average. Thus one might argue that education contributes to the observed differences between men and women in self-reported health. Studies have shown that education is one of the most important contributors to gender inequalities in health [22]. There are also several other explanations for such observed differences: economic dependence, employment, marital status, family position and family demands are among the factors found to contribute to gender differences in self-rated health [25,26]. However, it is argued that diversity in life style is not the most important reason for gender differences in social inequalities in health [27].

We found that women reported having significantly more chronic medical conditions. Studies from some other developing and transitional countries have yielded similar results, with some exceptions [28,29]. For instance, a Syrian study identified gender-specific determinants of poor self-rated health including being married, low socioeconomic status, and not having social support for women; and smoking, and low physical activity for men [28]. However, the present study showed that married men and particularly married women were worse off (in terms of health) than their single counterparts. One might argue that the married were merely older than the single, and being older implies worse health.

In addition to gender, the results from the present study clearly indicate that age has an independent effect on self-reported health. It is therefore argued that age, sex and social class make distinct contributions to specific morbidities and should be recognized as a transparent and robust approach to the assessment of morbidity-based inequality [30].

Finally, the limitations of this study should be considered in interpreting the results. The design was cross sectional and therefore could not indicate whether education really causes inequality in self-rated health or whether existing inequality due to other factors causes poorer health, and this in turn leads to lack of success in appropriate education. Furthermore, we used binary logistic regression analysis and dichotomized a 5-level ordinal scale to yield 'good or better than good' and 'less than good' self-rated health. Thus, excellent, very good and good self-ratings of health (for example) are assumed to be the same, but in fact they are not. There are other ways of analyzing such data and tackling this problem, for example by multinomial regression analysis, where ordinal data can be used without collapsing categories. However, each of these approaches to analysis has its own limitations. The study did not collect data on other measures such as health behaviors (diet, exercise, smoking, etc.) or measures such as blood pressure or body mass index. The contributions of these variables to self-rated health remain unknown. Collecting such data is recommended for future studies. We used years of formal education as a measure of socioeconomic position; it would be useful if reliable data on income could be collected for future investigations on the topic.

## Conclusion

The findings from this investigation provide further evidence for the education-related health inequalities. They suggest that health inequalities in developing countries such as Iran need to be addressed and policies for tackling the problem should be considered. In this respect, less well-educated people and women should be seen as the first target populations. It seems that although expanding the educational system might help the state to provide more educational options, it is also necessary to be sure that equal opportunities and access to quality education are provided for those from lower socioeconomic backgrounds; otherwise the current situation might cost the government more in the long term because of poor health in disadvantaged groups.

## Competing interests

The authors declare that they have no competing interests.

## Authors' contributions

All authors contributed to the study design and data collection. MV contributed to the data entry and analysis, AG contributed to writing of the first draft and AM supervised the study, analyzed the data and wrote the final manuscript. All authors read and approved the paper.
